# Editorial: Mobile assisted language learning: developments, affordances, and solutions

**DOI:** 10.3389/fpsyg.2023.1293483

**Published:** 2023-09-28

**Authors:** Rui Li, Di Zou, Barry Lee Reynolds, Boris Vazquez-Calvo

**Affiliations:** ^1^School of Foreign Languages, Hunan University, Changsha, Hunan, China; ^2^Department of English Language Education, The Education University of Hong Kong, Hong Kong, China; ^3^Faculty of Education, University of Macau, Macau, China; ^4^Centre for Cognitive and Brain Sciences, University of Macau, Macau, China; ^5^Department of Language Education, Faculty of Educational Sciences, University of Málaga, Málaga, Spain

**Keywords:** mobile assisted language learning (MALL), evidence-based applied linguistics, learner affect, pedagogical impact, computer assisted language learning (CALL)

The explosion of mobile technologies has sparked a growing academic interest with numerous research publications on mobile-assisted language learning (MALL).

MALL refers to the use smartphones, tablets wearable devices and other portable tools for language learning and teaching (Zhang and Zou, 2020; Li, 2022; Soyoof et al., 2023). These technologies are probed for their potential in creating portable, connective, context-sensitive, location-aware, multifunctional and ubiquitous learning environments. MALL tools have been demonstrated to bolster FL/L2 learners' skills, enabling learners to overcome spatial and temporal constraints with low-cost and high-quality personalized education. In other words, learners obtain a flexible learning experience and learning materials from anywhere and at any time, while actively engaged in self-paced or collaborative learning activities (Shadiev et al., 2020; Li, 2023a,b).

While the benefits of MALL are well-recognized, there exists a need for a deeper exploration into its latest developments, affordances, and solutions. These encompass social, contextualized, gamified affordances, interactive components, user perceptions, implications, and novel technological innovations. This Research Topic spotlights the most recent advancements and solutions in MALL. It contains seven research articles, which can be categorized in three broad categories:

1. Pedagogical impact: four articles utilizing quasi-experiments explore the educational effects of MALL tools.
2. Learner affections: two articles investigated foreign language learners' affections toward the use of MALL.
3. Teacher perceptions: the remaining one article focused on teachers' perceptions of MALL with a particular eye on identity reconstruction, attitude toward MALL, and some coping strategies for encountered difficulties.

Concerning the pedagogical impact, researchers normally adopted the quasi-experiment design to compare the pedagogical effects between participants using the MALL and those using the traditional approach. Xodabande and Boroughani argued that mobile-assisted focus on forms was found to be effective in both receptive and productive vocabulary learning. Similarly, Liu et al. examined the effects of mobile reading materials on children's vocabulary learning and posited that MALL could facilitate their development of vocabulary sizes rather than lexical diversity. Besides vocabulary learning, two articles focused on the use of MALL for learners' speaking skill development. Cai and Zhang explored the effects of mobile-supervised question-driven collaborative dialogues on learners' speaking strategies and performance and confirmed the pedagogical affordances of MALL for speaking performance and strategy use. Likewise, Liu also maintained that MALL could reduce learner speaking anxiety and enhance learning experience.

The second strand explored learner affections using questionnaire surveys. Hu et al. gave rise to revised technology acceptance model (TAM) and modeled MALL adoption with the integration of learner affective perceptions and flow experience. Yu et al. surveyed learning anxiety of MALL among Chinese rural high school students and claimed their learning anxiety was affected by the interplay of students' individual differences, teachers and environments.

Lastly, Huang et al. used semi-structured interviews to delve into English teachers' perspectives on MALL, exploring their attitudes, identity formation, and coping strategies. Results suggest that technical support and sustainable teacher professional development be warranted in the future research.

The papers published in this Research Topic foster fruitful dialogue between second language acquisition (SLA) and computer assisted language learning (CALL), which could not only contribute to the growing body of state-of-the-art knowledge on MALL, but also serve as a pathfinder for future SLA and CALL researchers to build theoretical and/or practical frameworks of the subject matter.

## Author contributions

RL: Conceptualization, Writing—original draft. DZ: Writing—original draft, Writing—review and editing. BR: Writing—original draft, Writing—review and editing. BV-C: Writing—original draft, Writing—review and editing.

## References

[B1] Li R. (2022). Effects of mobile-assisted language learning on EFL/ESL reading comprehension. Educ. Technol. Soc. 25, 15–29.

[B2] Li R. (2023a). Effects of mobile-assisted language learning on EFL learners' listening skill development. Educ. Technol. Soc. 26, 36–49.

[B3] Li R. (2023b). Effects of mobile-assisted language learning on foreign language learners' speaking skill development. Lang. Learn. Technol.

[B4] Shadiev R. Yang M. K. Reynolds B. L. Hwang W. Y. (2020). Improving English as a foreign language-learning performance using mobile devices in unfamiliar environments. Comput. Assis. Lang. Learn. 35, 2170–2200. 10.1080/09588221.2020.1868533

[B5] Soyoof A. Reynolds B. L. Vazquez-Calvo B. McLay K. (2023). Informal digital learning of English (IDLE): a scoping review of what has been done and a look towards what is to come. Comput. Assis. Lang. Learn. 36, 608–640. 10.1080/09588221.2021.1936562

[B6] Zhang R. F. Zou D. (2020). Influential factors of working adults' perceptions of mobile-assisted vocabulary learning with multimedia annotations. Int. J. Mob. Learn. Org. 14, 533–548. 10.1504/IJMLO.2020.110798

